# V‐Y Tendon Plasty for Reconstruction of Chronic Achilles Tendon Rupture: A Medium‐term and Long‐term Follow‐up

**DOI:** 10.1111/os.12429

**Published:** 2019-02-27

**Authors:** Yang‐jing Lin, Xiao‐jun Duan, Liu Yang

**Affiliations:** ^1^ Center for Joint Surgery, Southwest Hospital Third Military Medical University (Army Medical University) Chongqing China

**Keywords:** Achilles tendon, Chronic, Rupture, V‐Y tendon plasty

## Abstract

**Objective:**

To evaluate the surgical results of V‐Y tendon plasty in the treatment of chronic Achilles tendon rupture during medium and long‐term follow‐up.

**Methods:**

Between June 2005 and September 2017, 20 patients with chronic Achilles tendon rupture underwent V‐Y tendon plasty in our hospital. The mean injury‐to‐surgery time was 20.4 weeks (range, 4–96 weeks). The Matles test and an improved Thompson test was used to examine Achilles tendon rupture. These patients were not able to stand on the tiptoes of the injured lower extremity. X‐ray tests ruled out the chance of fracture and were used to examine the Kager triangle. MRI was used to confirm the final diagnosis. The function of the ankle and the foot was assessed using the American Orthopaedic Foot & Ankle Society (AOFAS) score and the Achilles Tendon Total Rupture Score (ATRS). V‐Y tendon plasty for Achilles tendon reconstruction was performed. A below‐knee cast was used to keep the ankle in plantar flexion (up to 20°) for 4 weeks. Non‐weight‐bearing exercise was allowed with crutches. After 4 weeks, partial weight‐bearing was allowed. Physical exercises were performed under rehabilitation guidelines. At 10–12 weeks postoperation, patients began to return to daily life activity levels without restrictions. Preoperative and postoperative MRI was obtained.

**Results:**

The mean follow‐up period was 32.8 months. The mean operative gap of the Achilles tendon after debridement was 5 cm (range, 4–9 cm), with 85% of the gap less than or equal to 6 cm. The mean AOFAS score increased from 59.25 ± 12.28 preoperatively to 96.55 ± 3.75 at final follow‐up (*P* < 0.05). The mean ATRS score increased from 39.55 ± 14.21 preoperatively to 94.05 ± 4.89 at final follow‐up (*P* < 0.05). All patients had no recurrent Achilles tendon rupture during the follow up. No patient had developed serious complications, such as sural nerve injury or deep vein thrombosis. Patients were able to return to daily life activity levels without restrictions. At the latest follow‐up, all patients were able to perform repetitive single heel rise on the involved limb, and to walk without a visible limp. All of the postoperative MRI showed the continuity of the Achilles tendon with no signs of cysts or inflammation, indicating perfect healing at the final follow‐up.

**Conclusions:**

V‐Y tendon plasty can be used in most cases of chronic Achilles tendon rupture. It yields satisfactory functional results and low complication rates. The advantage of this procedure is that it is an easy and economic method without the need for expensive synthetic implants. V‐Y tendon plasty should be considered an acceptable first‐choice treatment.

## Introduction

The Achilles tendon is attached to the triceps surae at one end and the calcaneus at the other. It plays a key role in energy transfer, energy storage, and energy release during foot and ankle locomotion. The Achilles tendon can be subjected to incremental loads of up to 12 times a person's weight during running^1^. Despite its strength, the Achilles tendon is one of the most common tendons to spontaneously rupture^2^. More and more people are participating in sports, so the incidence of sport injuries is also increasing. Achilles tendon rupture is one of the most common lower extremity injuries. Most ruptures occur playing football, basketball, and badminton. Clinically, even though acute Achilles tendon rupture is usually not difficultly to diagnose and cure with an experienced surgeon, approximately 20%–25% of acute injuries are neglected, leading to chronic Achilles tendon rupture^3^. Chronic Achilles tendon rupture is diagnosed if the rupture occurs within 4–6 weeks after injury (misdiagnosis or no effective treatment)^4^. The clinical manifestations of chronic Achilles tendon rupture include chronic pain, claudication, and weak or absent heel rise. It seriously affects these patients’ daily lives.

The treatment of chronic Achilles tendon rupture is different from the treatment of acute Achilles tendon rupture. Most patients can be effectively treated with end‐to‐end sutures for acute Achilles tendon rupture and attain a satisfactory effect after rehabilitation. Retraction of the tendon ends can often be observed in chronic Achilles tendon rupture. Sometimes the gap in chronic Achilles tendon rupture is bridged by fibro‐adipose scar tissue. Fibro‐adipose scar tissue are not normal tendon fibers and can lead to ankle weakness and gait disturbances^5^, ^6^, ^7^, so we need to remove the scar tissue completely. However, after excising the intervening scar tissue, a gap (more than 2 cm) is usually left and it is difficult to repaired in an end‐to‐end manner^6^, ^8^. Restoration of enough length and tension is vital to the functional recovery of Achilles tendons. Sometimes it is very difficult to perform the reconstruction of chronic Achilles tendon ruptures. Therefore, the treatment of chronic Achilles tendon ruptures is often a challenge for orthopedic surgeons.

Various methods have been adopted to achieve the desired effects to repair the gap, such as V‐Y tendon plasty, gastrocnemius fascial turndown flap, tendon transfer (flexor hallucis longus tendon transfer and flexor digitorum longus tendon transfer^9^), allograft reconstruction, autograft reconstruction (semitendinosus tendon graft^10^, fibularis brevis, and fibularis longus muscles^11^), synthetic graft augmentation (ligament advanced reinforcement system^12^, bio‐absorbable synthetic graft^13^), and biologic matrix augmentation^14^. The reconstruction of chronic Achilles tendon rupture is complex, and it might affect the choice of procedures. There is no standard treatment for chronic Achilles tendon rupture, especially with large defects^5^. We have no recognized strategy for surgical treatments for chronic Achilles tendon rupture. No evidence of optimal treatments for chronic Achilles tendon rupture is shown in evidence‐based medicine. Sometimes it is difficult to choose a method for optimal therapy of chronic Achilles tendon among the various methods. Many studies reveal postoperative and follow‐up results for gastrocnemius fascial turndown flap and tendon transfer. However, medium and long‐term follow‐up results for pure V‐Y tendon plasty have seldom been reported.

The purpose of this study is to evaluate clinical outcomes of our treatments based on V‐Y tendon plasty for chronic Achilles tendon reconstruction in medium and long‐term follow up. The goal of this study is also to better understand the treatment of chronic Achilles tendon rupture by reviewing the clinical outcomes of V‐Y tendon plasty for Achilles tendon reconstruction.

## Materials and Methods

### *Patient Selection*

Inclusion criteria: (i) Achilles tendon rupture was diagnosed more than 4 weeks after injury; and (ii) patients with Achilles tendon rupture had undergone V‐Y tendon plasty. Exclusion criteria: (i) open rupture of Achilles tendon; and (ii) concomitant diseases with local infection, neurovascular injury, or lower extremity fracture.

### *Patients’ Information*

**Figure 1:**
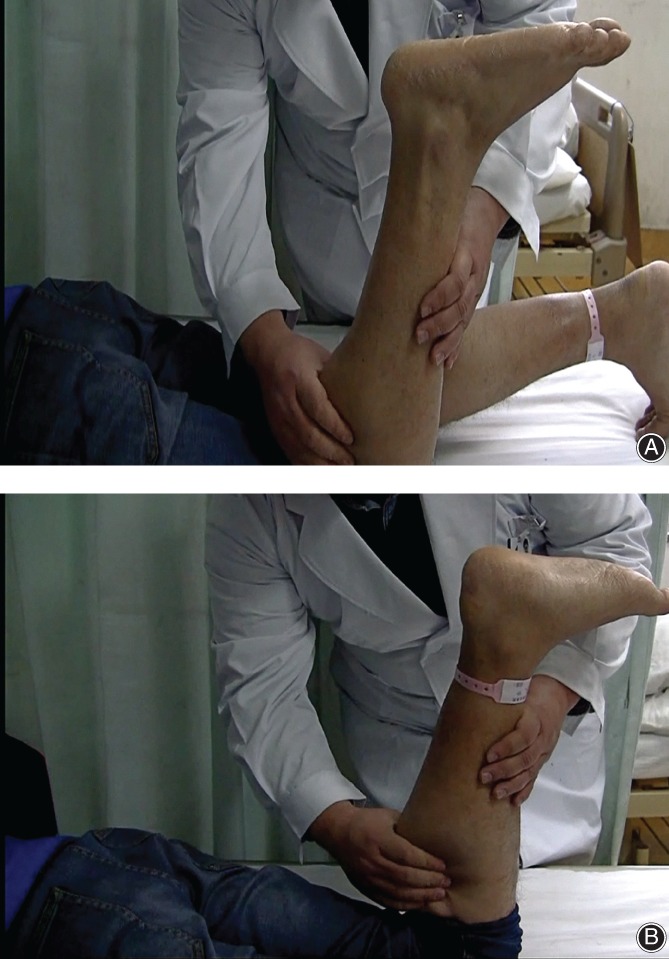
(A) Improved Thompson test. The patient was placed prone on the examining table, bending 90° at the knee and squeezing the gastrocnemius muscle. The positive sign was no plantar flexion. (B) The normal foot can perform plantar flexion.

**Figure 2:**
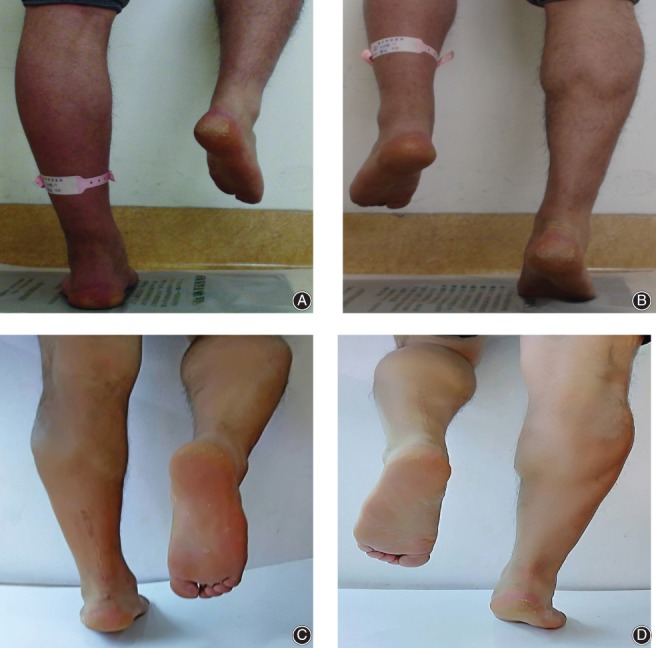
(A) The patient was not able to stand on the tiptoes of the injured lower extremity. (B) The patient was able to stand on the tiptoes of the other lower extremity. (C) The patient was able to stand on the tiptoes of the injured lower extremity at the latest follow‐up. (D) The patient was able to stand on the tiptoes of the other lower extremity at the latest follow‐up.

**Figure 3:**
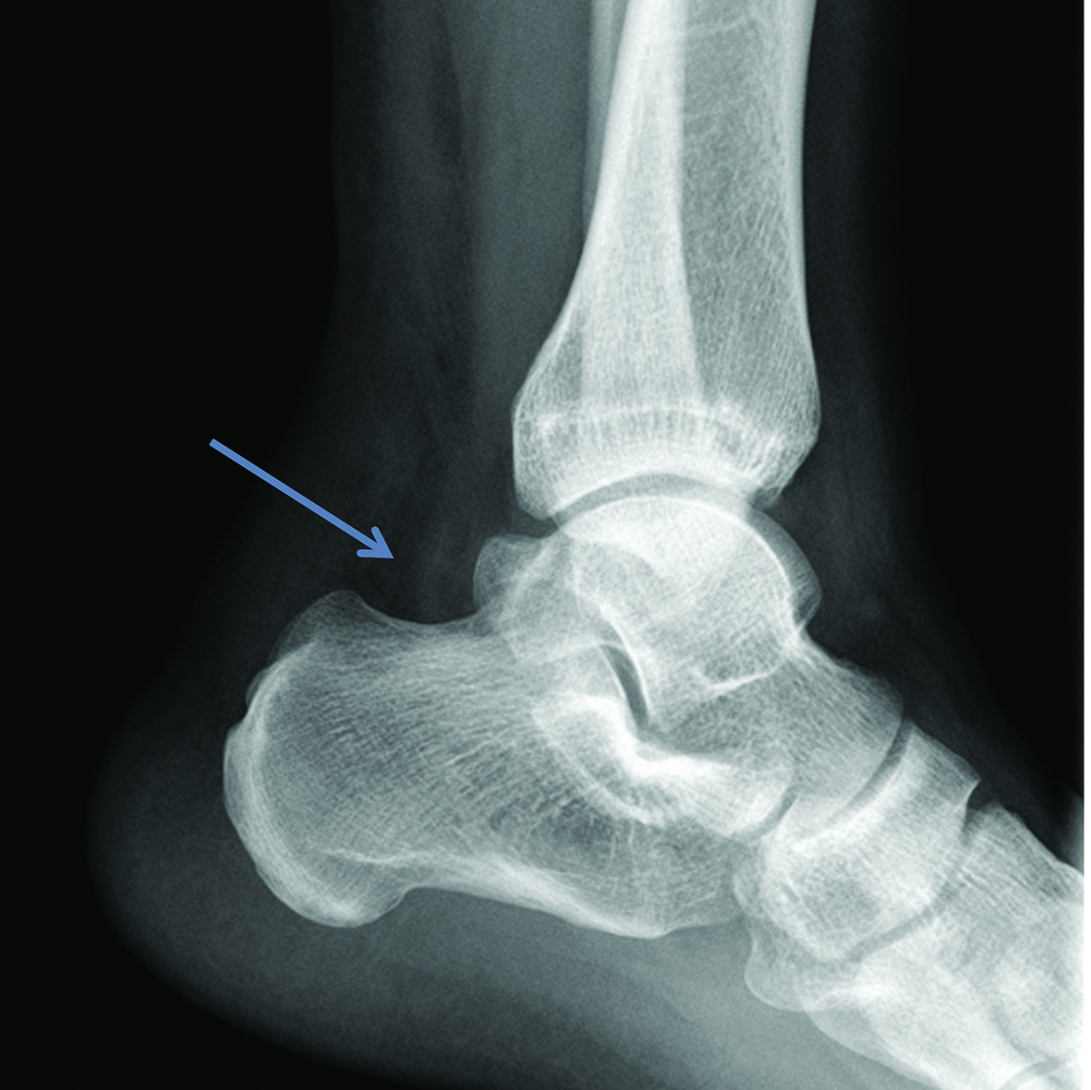
The borders of the Kager triangle became distorted (arrow).

**Figure 4:**
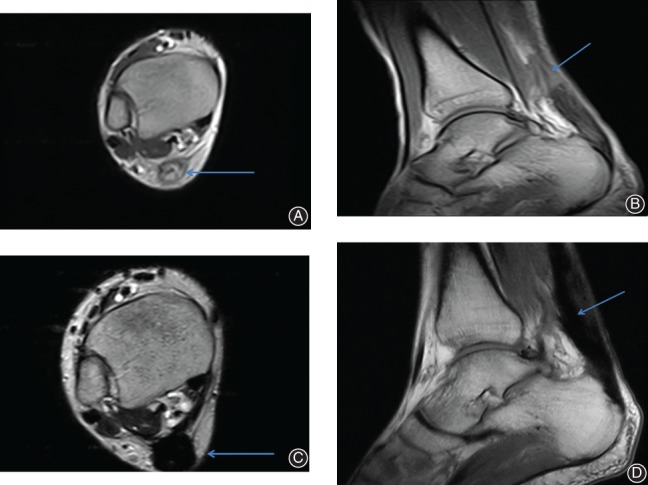
A 39‐year‐old man. The interval from rupture to surgery was 8 weeks. (A, B) MRI showed chronic Achilles tendon rupture in transaxial and sagittal planes (arrow). (C, D) MRI showed the continuity of the Achilles tendon with no signs of cysts or inflammation in transaxial and sagittal planes at the latest follow‐up (arrow).

Between June 2005 and September 2017, 20 patients with chronic Achilles tendon rupture underwent V‐Y tendon plasty for Achilles tendon reconstruction in our hospital. The mean injury‐to‐surgery time was 20.4 weeks (range, 4–96 weeks). In our study, 14 patients were misdiagnosed after the first injury; among the other 6 patients with correct first‐time diagnosis, 5 patients suffered failure of conservative treatment and 1 patient suffered recurrent rupture (Table 1). The Matles test^15^ and an improved Thompson test was used to examine Achilles tendon rupture (Fig. 1). None of the patients were able to stand on the tiptoes of the injured lower extremity (Fig. 2). X‐ray tests ruled out the chance of fracture and were used to examine the Kager triangle (Fig. 3). With an Achilles tendon rupture, the Kager triangle may appear deformed (such as the triangle disappearing or higher density) on a lateral radiograph^16^. MRI was used to confirm the final clinical diagnosis in trans axial and sagittal planes (Fig. 4A, B).

**Table 1:** Characteristics of patient

Characteristics	Patients (*n* = 20)
Sex	
Male	16
Female	4
Age (years)	38.5 (20–71)*
Side (cases)	
Left	8
Right	12
Reasons for chronic rupture (cases)	
Neglect	14
Failed treatment	6
Time from injury to surgery (weeks)	20.4 (4–96)*
Gap after debridement (cm)	5 (4–9)*
Follow‐up period (months)	32.8 (12–68)*

* The value was given as the mean, with the range in parentheses.

Of all these patients, 16 patients were male and 4 patients were female, with the mean age of 38.5 years (range, 20–71 years) at surgery. Eight cases were left chronic Achilles tendon ruptures and the other 12 cases were right chronic Achilles tendon ruptures (Table 1). The function of the ankle and the foot in the involved patients were evaluated by AOFAS ankle–hindfoot score and ATRS score. In addition, postoperative MRI of the Achilles tendon was obtained.

### *Surgical Technique*

**Figure 5:**
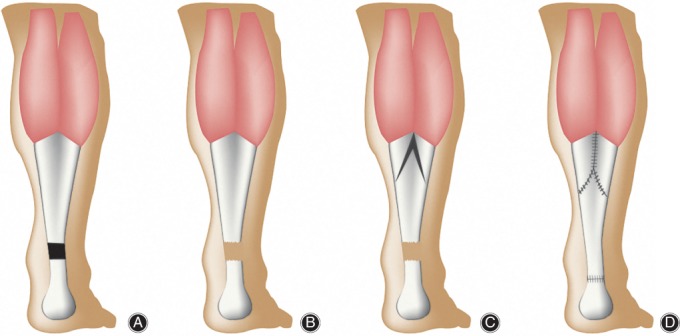
(A) Surgical diagrams of V‐Y tendon plasty. Chronic Achilles tendon rupture was bridged by fibro‐adipose scar tissue. (B) Tendinopathic tendons were debrided. (C) Design of the V flap. (D) End‐to‐end anastomosis and “Y” is sutured.

**Figure 6:**
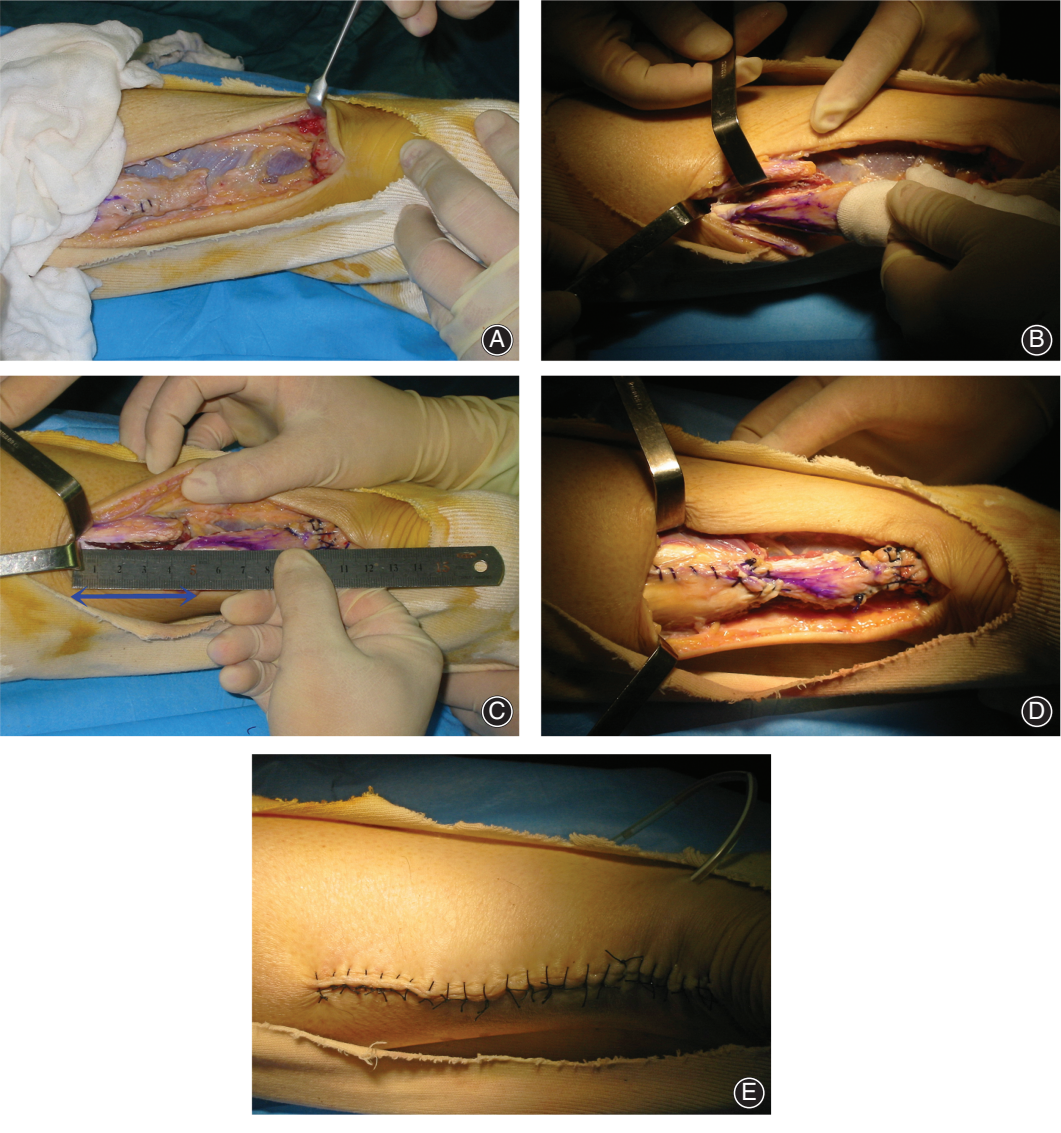
A 41‐year‐old man. The interval from rupture to surgery was 4 weeks. (A) All the tendinopathic tendons were then sharply debrided back to healthy tendons. (B) The V‐shaped part was slowly torn and pulled towards the distal stumps with caution. (C) The repaired length (double‐headed arrow) was 5 cm. (D) The V‐Y tendon plasty was completed. (E) The skin was closed.

The patient was placed prone on the operating table under anesthesia of lumbar plexus‐sciatic nerve block and general anesthetic. A thigh tourniquet was used. The heels of the patient were positioned over the edge of the operating table. Then V‐Y tendon plasty for Achilles tendon reconstruction was performed (Fig. 5). A posterior approach was made over the position of the rupture to minimize or eliminate incision‐related complications. The rupture position of the Achilles tendon was adequately exposed. The surrounding superficial fascial and paratenon layer were carefully preserved to simplify the wound closure. This was an important step in preventing infection. The scar of the fibro‐adipose pseudotendon was identified within the gap. All the tendinopathic tendons were then sharply debrided back to healthy tendon (Fig. 6). After debridement, the gap was measured between the tendon stumps. When the tendon stumps had enough integrity of the Achilles tendon, ruptures of the gap less than 2 cm could be repaired directly using Krakow method, while gaps greater than 2 cm could be addressed through V‐Y tendon plasty. For the V‐Y tendon plasty, the V‐shaped part was designed in the gastrocnemius aponeurosis; the arms of the V should be at least one and a half times longer than the size of the defect in the Achilles tendon; then the V‐shaped part should be slowly torn and pulled towards the distal stumps with caution until it can bridge the gap (Fig. 6). The foot–ankle was held in an appropriate position during the operation. A 1/0 non‐absorbable suture was used to repair the V and the distal stumps together with the Krakow method. A 2/0 absorbable suture was used to suture the proximal incision in a Y configuration. After reconstruction of the Achilles tendon rupture, the paratenon layer was sutured with 2/0 absorbable sutures, then the skin was closed in layers (Fig. 6).

### *Postoperative Treatment*

A below‐knee cast was used to keep the ankle in plantar flexion (up to 20°) for 4 weeks. Non‐weight‐bearing exercise was allowed with crutches. After 4 weeks, partial weight‐bearing was allowed. Physical exercises were performed under rehabilitation guidelines. At 10–12 weeks postoperation, patients began to return to daily life activity levels without restrictions.

### *Postoperative Evaluation*

MRI and physical examination were conducted during follow up. The postoperative Achilles tendon function was evaluated by ankle strength, pain, and ankle range of motion. The patient was examined performing repetitive single‐limb heel rises. Surgery‐related complications were recorded, such as infection and wound healing problems. At the latest follow‐up, AOFAS scores, and ATRS scores were evaluated.

### *Statistical Analysis*

Preoperative and postoperative AOFAS and ATRS scores were recorded and reevaluated. Comparisons between the preoperative and postoperative AOFAS and ATRS scores were compared using paired‐sample *t*‐tests in the Statistical Package for the Social Sciences (version 18.0; SPSS, IBM, Chicago, IL, USA). Significance level was set at *P* < 0.05.

## Results

### *General Results*

**Figure 7:**
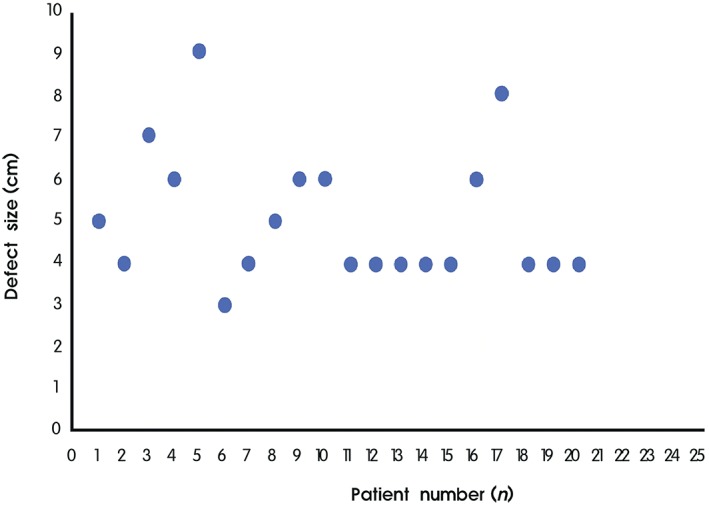
Scatter diagram showed that the majority of the defect size was less than or equal to 6 cm.

The mean operative gap of the Achilles tendon after debridement was 5 cm (range, 4–9 cm). Eighty‐five per cent of the gap was less than or equal to 6 cm (Fig. 7). The mean follow‐up period was 32.8 months (range, 12–68 months; Table 1). All the postoperative MRI showed the continuity of the Achilles tendon with no signs of cysts or inflammation, indicating perfect healing (Fig. 4).

### *Complications in Mid‐term to Long‐term Follow‐up*

All patients had no recurrent Achilles tendon rupture during the follow up. No patient had developed serious complications, such as sural nerve injury or deep vein thrombosis. Superficial incision infection was recorded in 1 case. The infection healed following debridement and using antibiotics.

### *Evaluation in Activity Level*

No patient was limited when walking on uneven surfaces and walking quickly up stairs. None of the patients were limited in performing activities, including running and jumping. They could achieve daily life activity levels without restrictions. At the latest follow up, all patients were able to perform repetitive single heel rise on the involved limb (Fig. 2) and to walk without a visible limp.

### *Functional Score Results*

The mean AOFAS score increased from 59.25 ± 12.28 (range, 40–75) preoperatively to 96.55 ± 3.75 (range, 90–100) at final follow‐up (*P* < 0.05). The mean ATRS also showed significant improvement, with the preoperative score of 39.55 ± 14.21 (range, 20–72) and the latest follow‐up score of 94.05 ± 4.89 (range, 86–100) (*P* < 0.05, Table 2).

**Table 2:** AOFAS ankle–hindfoot scores and Achilles tendon total rupture score (mean ± SD)

Scores	Preoperation	Postoperation	*P‐*value
AOFAS	59.25 ± 12.28	96.55 ± 3.75	0.00*
ATRS	39.55 ± 14.21	94.05 ± 4.89	0.00*

* Significance level was set as *P* < 0.05.

AOFAS, American Orthopaedic Foot & Ankle Society score; ATRS, Achilles Tendon Total Rupture Score; SD, standard deviation.

## Discussion

Chronic Achilles tendon rupture can be diagnosed at 4–6 weeks after injury if left untreated^17^. The typical symptoms reported include pain, decreased strength, fatigue, and ankle stiffness. The defect from the rupture will be bridged by scar tissue after the first week of injury. A palpable large gap between the rupture ends can be commonly observed after debriding scar tissue. For most orthopedic surgeons, chronic Achilles tendon ruptures with large gaps are a challenge to treat. Various methods of reconstruction are used in clinical treatment for Achilles tendons, such as V‐Y tendon plasty, gastrocnemius fascial turndown flap, tendon transfer, allograft reconstruction, autograft reconstruction, synthetic graft augmentation, and biologic matrix augmentation. V‐Y tendon plasty is an effective and economic method in the classical operation. V‐Y tendon plasty can be performed in cases of medium and large‐sized (more than 2 cm) defects. It can restore full strength of the Achilles tendon and, thus, improve the activity level of patients^18^. Our results in the study support the surgery operation.

V‐Y tendon plasty was first reported by Abraham and Pankovich as an effective treatment for chronic Achilles tendon ruptures^19^. We performed V‐Y tendon plasty in 20 patients with chronic Achilles tendon rupture. In these cases, significant improvement in the AOFAS and ATRS was observed at latest follow‐up and no reruptures had occurred. At the latest follow‐up, all patients were able to perform repetitive single heel rise on the involved limb and to walk without visible limp. No severe wound healing complication was observed. The mean defect size in our study was 5 cm (range, 4–9 cm) and the maximal gap was 9 cm. Ahmad *et al*. considered a gap more than 6 cm in chronic Achilles tendon rupture to be a big challenge to surgeons^5^. McClelland deemed that V‐Y tendon plasty could attain satisfactory results for a gap measuring more than 6 cm in length. The size of the chronic Achilles tendon rupture defect is a vital factor in the operation^20^. In our result, 85% of gaps were less than or equal to 6 cm. Sixty‐five per cent of gaps were less than or equal to 5 cm. V‐Y tendon plasty can be easily used in most cases of chronic Achilles tendon rupture. In our study, a gap up to 6 cm could also be repaired. All patients regained tendon strength and could perform repetitive single heel rise. We were also able to achieve satisfactory results when the maximal gap was 9 cm. No other complex technique was used in our V‐Y tendon plasty, such as tendon transfer and harvesting autologous tendon. We only followed a strict method of classical V‐Y tendon plasty. Guclu *et al*. reported that V‐Y tendon plasty with fascia turndown in chronic Achilles tendon rupture could restore functional length^21^. In our study, not combining the V‐Y tendon plasty with the fascia turndown could also restore functional length. In our view, V‐Y tendon plasty is an effective and easy method for reconstruction of chronic Achilles tendon rupture. It can be easily used in most cases of chronic Achilles tendon rupture.

A gap greater than 2 cm can also be addressed through gastrocnemius fascial turndown flap in chronic Achilles tendon rupture treatment. It was also a useful technique in repairing chronic Achilles tendon ruptures with great defects. However, most patients reported significant discomfort and weakness at the tendon following gastrocnemius fascial turndown flap^5^. Takao *et al*. reported strength deficits of 9%–23% postoperatively following treatment with gastrocnemius fascial turndown flap^22^. The reason might be that the strength of the gastrocnemius fascial flap was less than the tendon strength. The tendon of V‐Y tendon plasty could be revascularized from soleus muscle so that the Achilles tendon rupture could heal faster. In comparison, gastrocnemius fascial turndown flap cannot directly achieve revascularization of soleus muscle. Therefore, the rupture may heal more slowly. In our opinion, gastrocnemius fascial turndown flap should be not the first choice.

Flexor hallucis longus (FHL) transfer has developed to become a good treatment for chronic Achilles tendon rupture. However, this method may reduce the function of halluces; therefore, it should not be routinely used for young patients. According to Wegrzyn *et al*., patients who were treated with FHL transfer participated in less sports than pre‐injury^23^. Besides, there would be a rather long process of interface structure reconstruction between bone and tendon. Wegrzyn *et al*. report that patients treated with FHL transfer returned to sports activities after an average of 10 months^23^. Like other methods (e.g. allograft reconstruction, autograft reconstruction, and synthetic graft augmentation), flexor hallucis longus transfer requires synthetic materials for augmentation. These methods are not economic alternatives and do not reduce the burden of health‐care costs for society. V‐Y tendon plasty can be performed without expensive synthetic implants and allograft. It is an economic choice for patients with chronic Achilles tendon rupture.

The limitation of the current study was the small number of cases. It was a retrospective study. In addition, we undertook no isokinetic strength analysis of the cases. Future studies with larger numbers of cases and longer follow‐up time could provide stronger evidence for the use of V‐Y tendon plasty in the treatment of chronic Achilles tendon rupture.

### *Conclusions*

This study has shown that the gap size in chronic Achilles tendon rupture is typically less than or equal to 6 cm. V‐Y tendon plasty can be easily used in most cases of chronic Achilles tendon rupture. It yields satisfactory functional results and relatively low complication rates. This technique can bridge a large rupture gap, even if the presence of a gap as large as 9 cm. The advantage of this procedure is that it is an easy and economic method without the need for expensive synthetic implants. V‐Y tendon plasty should be considered an acceptable first‐choice treatment.
